# Cell image reconstruction using digital holography with an improved GS algorithm

**DOI:** 10.3389/fphys.2022.1040777

**Published:** 2022-10-31

**Authors:** Yuhao Jiang, Hongzhong Li, Yu Pang, Jiwei Ling, Hao Wang, Yuling Yang, Xinyu Li, Yin Tian, Xiuxin Wang

**Affiliations:** ^1^ Institute for Advanced Sciences, Chongqing University of Posts and Telecommunications, Chongqing, China; ^2^ Central Nervous System Drug Key Laboratory of Sichuan Province, Luzhou, China; ^3^ School of Civil Engineering, Guangdong Communication Polytechnic, Guangzhou, China; ^4^ Guangyang Bay Laboratory, Chongqing Institute for Brain and Intelligence, Chongqing, China; ^5^ Key Laboratory of Opto-technology and Intelligent Control, Ministry of Education, Lanzhou Jiaotong University, Lanzhou, China

**Keywords:** computer hologram, cell image, GS algorithm, phase reconstruction, reconstruction

## Abstract

Digital holography is an effective technology in image reconstruction as amplitude and phase information of cells can be acquired without any staining. In this paper, we propose a holographic technique with an improved Gerchberg-Saxton (GS) algorithm to reconstruct cell imaging based on phase reconstruction information. Comparative experiments are conducted on four specific models to investigate the effectiveness of the proposed method. The morphological parameters (such as shape, volume, and sphericity) of abnormal erythrocytes can be obtained by reconstructing cell hologram of urinary sediment. Notably, abnormal red blood cells can also be detected in mussy circumstances by the proposed method, owing to the significantly biophysical contrast (refractive index distribution and mass density) between two different cells. Therefore, this proposed method has a broad application prospect in cell image reconstruction and cell dynamic detection.

## Introduction

In the research field of biomedical and life sciences, researchers expect to capture the cell morphological shapes, dynamic characteristics, physiological parameters, interaction among cells, cell reactions to medicines and drug transport information by visual observation of biological cells in nutrient solution or under natural circumstances, which has an important role in early diagnosis and medicine design (Dixit and Cyr, 2003). Optical microscopy, which is regarded as a conventional medical imaging tool, is capable of magnifying images of minute object specimens with visible light. However, the conventional optical microscope can only examine the strength of the optical wave and achieve a two-dimensional, light intensity distribution map. Optical microscopy has difficulty capturing the three-dimensional spatial information of specimens. In addition, fluorescence microscopy demonstrated great performance in enhancing the contrast in cell images (Otuboah et al., 2019; Wen et al., 2020). Before observation using fluorescence microscopy, stains and dyes, such as rhoda mine, acridine orange, green fluorescent protein or other substances, are usually employed to mark biological cells to enhance the contrast in different biological specimens (Kricka and Fortina, 2009). The staining pretreatment process not only increases the complexity of cell imaging in operation but also greatly impacts cellular activity. Therefore, with the development of biomedical science, conventional cell imaging technologies have difficulty meeting the requirements of the dynamic observation and quantitative analysis of living cells (Ushenko et al., 2018; Borovkova et al., 2019; Ushenko et al., 2019; Peyvasteh et al., 2020).

Optical holography is a technology that adopts a photosensitive medium to record the optical wavefront information and that subsequently superimposes a second wavefront, which is referred to as a diffracted wave, to reconstruct the final image (Su et al., 2003; Wang et al., 2014; Lucchetta et al., 2015; Deng and Li, 2017). Digital holography is established by optical holography, dividing the procedures into recording and reconstruction (Demoli et al., 2019; Xia et al., 2019). Digital holography has drawn much attention from researchers as a cell imaging technique, owing to its advantageous characteristics of low production cost, rapid reconstruction speed, flexible recording and capturing of phase information of optical fields (Wang et al., 2018; Zhong et al., 2020). Digital holography utilizes photoelectric sensors (i.e., CCD or COMS) instead of a photosensitive medium to record the wavefront information, which is processed by computers. The light transmitting process is simulated by a computer based on diffraction propagation theory to quantitatively calculate the amplitude and phase distribution of the recorded object. Although existing optical instruments easily capture the light intensity data of high-frequency optical waves, the phase information is hard to collect or even lost in the course of recording. Notably, the optical wave phase has important morphological information with respect to the specimens and may contain up to 80% information of the imaged specimens. A phase retrieval algorithm has been reported to promote the speed of reconstructing images of X-rays, and this method is responsible for phase holograms in both the visible band and the non-visible band (Hennelly and Sheridan, 2003). Digital holographic techniques have been reported for dynamic estimation by analyzing the specimen shape and deformation analysis (Cui et al., 2010; Jin et al., 2017). Recently, digital holographic techniques also had been adopted for specific neural activity patterns (Adesnik and Abdeladim, 2021), monitoring cell characteristic variations in their density (Delikoyun et al., 2021), and cells migrating in a 3D environment (Hellesvik et al., 2020).

In this paper, we proposed a holographic technique to reconstruct cell imaging based on phase recovery information using an improved GS algorithm. The improved GS algorithm could optimize the initial phase based on local convergence theorem, and accomplish the reconstructed holographic image from contrapuntal phase information. We built four specific models to investigate the effectiveness of the proposed method. Comparative experiment results demonstrated that the proposed method is able to reconstruct phase images with a high signal-to-noise ratio, in which the edge and whole structural information has been reserved. The proposed method is utilized to detect abnormal red blood cells in urine sediment. Specifically, the improved GS algorithm enhances the quality of the phase-reconstructed holographic cell image and successfully detects abnormal red blood cells with distinct edges from the mussy circumstance. This method has great potential for practical biomedical applications in monitoring cell activities.

## Methods

### Digital holographic instruments

**FIGURE 1:**
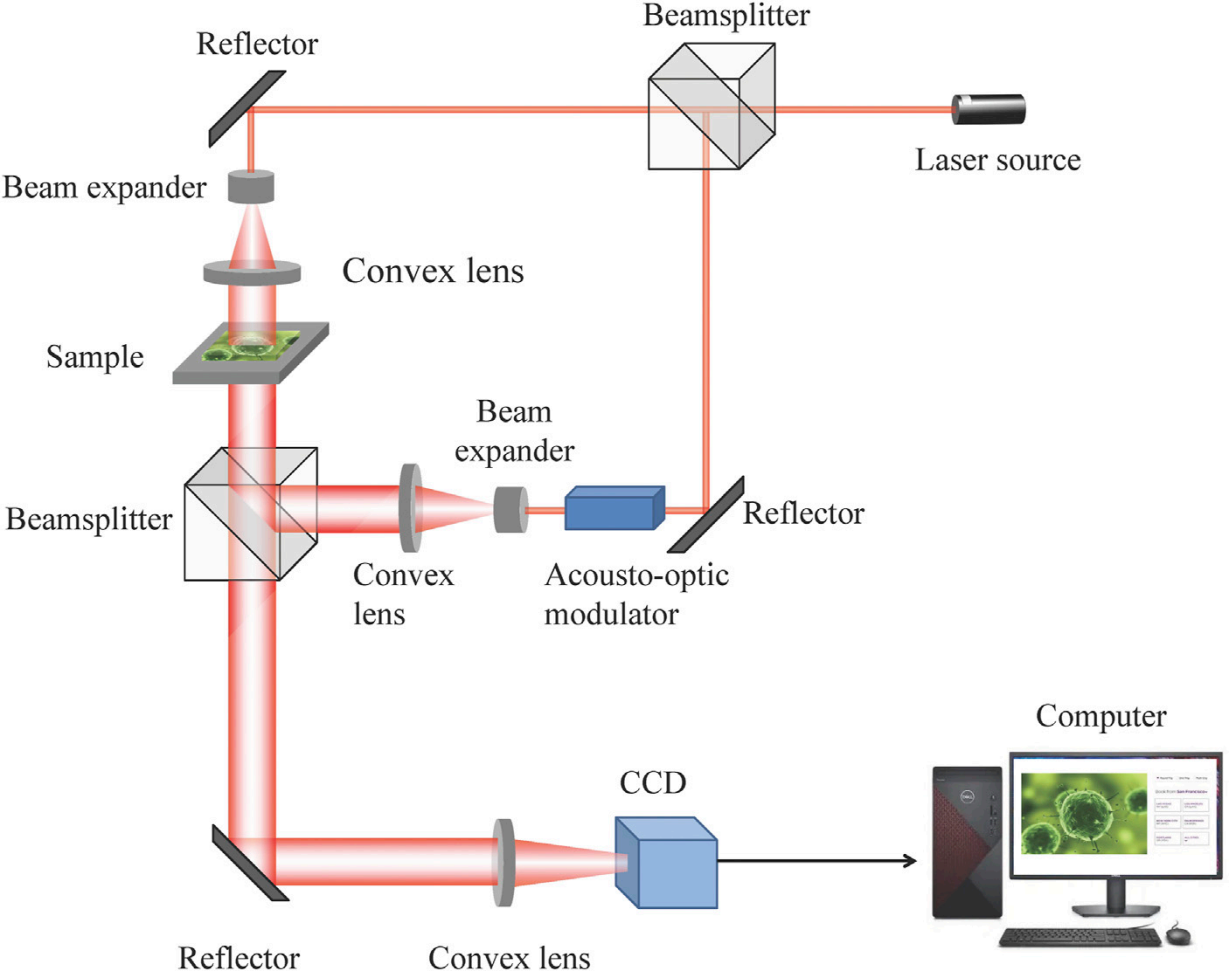
General setup of the holographic imaging measurement system.

In this section, Figure 1 shows the general setup of the holographic imaging measurement system for holographic information acquisition and object reconstruction, and the optical holography adopts optical interference and diffraction theories to establish the light path. The system is composed of a laser source, beamsplitter cube, lens, reflector and beam receiver CCD. The laser beam emitted by the laser source is split into two orthogonal laser beams *via* the beamsplitter cube, and the laser beams are expanded by a beam expander. The outgoing beams from the beam expander are then collimated by a convex lens, and the beams are regarded as plane waves. One incident plane wave is projected onto the sample object, and the other plane wave goes through the acoustic-optic modulator as the reference wave. The frequency of outgoing waves from the acoustic-optic modulator has changed. Then, the light wave projected by the sample object and reference wave transmit through different optical paths and then become incident on the CCD sensor, on which optical interference simultaneously occurs. The CCD sensor acquires the holographic data, which are saved by the computer. The digital holographic technique eliminates the laser source and complex light path structure with respect to optical holography. The digital holographic technique saves the interference pattern generated on the conventional sensor plane of optical holography as a digital image for simulation (Wang et al., 2019). By computing the complex amplitude map of the optical wave, the phase information or amplitude information can be coded, and the encoding function is adopted to depict the diffracted propagation of the object projected wave from the holographic plane to the object plane. The digital holographic map is reconstructed by computer holography (Schnars and Jüptner, 2002).

### Holographic imaging algorithm

The holographic image is reconstructed in the steps of computer holography. First, we conduct a discrete sampling operation on the optical images to obtain a discrete digital image. A random initial phase distribution of the input optical field corresponding to the amplitude of the sampled image is generated for iterative process. Second, we conduct Fresnel or Fourier transformation on the discrete optical field image. Third, we use an optimal encoding scheme to transform the amplitude and phase information into the optimal transmittance expression function. Last, the holographic image is reconstructed through iterative calculations based on the encoding function.

During computer holographic image reconstruction, we simulate the holographic imaging process and optical diffraction theory by computer calculation based on scalar diffraction theory, which is responsible for describing optical propagation. In computer holography, z defines the distance between the diffractive plane and the observed plane, and U(x,y,0) and U(x,y,z) demonstrate the complex amplitudes of the optical wave on the diffractive plane and observed plane, respectively. The Fourier spectra regarding the complex amplitudes of the optical wave, diffractive plane and observed plane are $F_{0}\left(f_{x},f_{y}\right)$ and $F_{z}\left(f_{x},f_{y}\right)$ . Given that the spectrum of the optical wave on the observed plane is known, the inverse Fourier transformation is conducted to calculate the amplitude on the observed plane, and the calculating function can be expressed as follows:

$$U\left(x,y,z\right)=\int_{-\infty }^{+\infty }\int_{-\infty }^{+\infty }F_{z}\left(f_{x},f_{y}\right)e^{j2\pi \left(f_{x}x+f_{y}y\right)}df_{x}df_{y}$$ (1)

where $\left(f_{x},f_{y}\right)$ demonstrates spatial frequency of simple harmonic plane wave and $e^{j2\pi \left(f_{x}x+f_{y}y\right)}$ demonstrates complex amplitude distribution of simple harmonic plane wave in (x, y) coordinates. Next, the correlation between $F_{0}\left(f_{x},f_{y}\right)$ and $F_{z}\left(f_{x},f_{y}\right)$ is calculated by substituting Eq. 1 into the Helmholtz equation (Liu and Scott, 1987), which is expressed as follows:

$$F_{z}\left(f_{x},f_{y}\right)=F_{0}\left(f_{x},f_{y}\right)e^{j\frac{2\pi }{\lambda }z\sqrt{1-{\left(\lambda f_{x}\right)}^{2}-{\left(\lambda f_{y}\right)}^{2}}}$$ (2)

Then, we obtain the following equation.

$$U\left(x,y,z\right)=IFFT\langle FFT\left\{U\left(x,y,0\right)\right\}e^{j\frac{2\pi }{\lambda }z\sqrt{1-{\left(\lambda f_{x}\right)}^{2}-{\left(\lambda f_{y}\right)}^{2}}}\rangle$$ (3)

where FFT demonstrates the Fourier transformation and IFFT demonstrates the inverse Fourier transformation.

**FIGURE 2:**
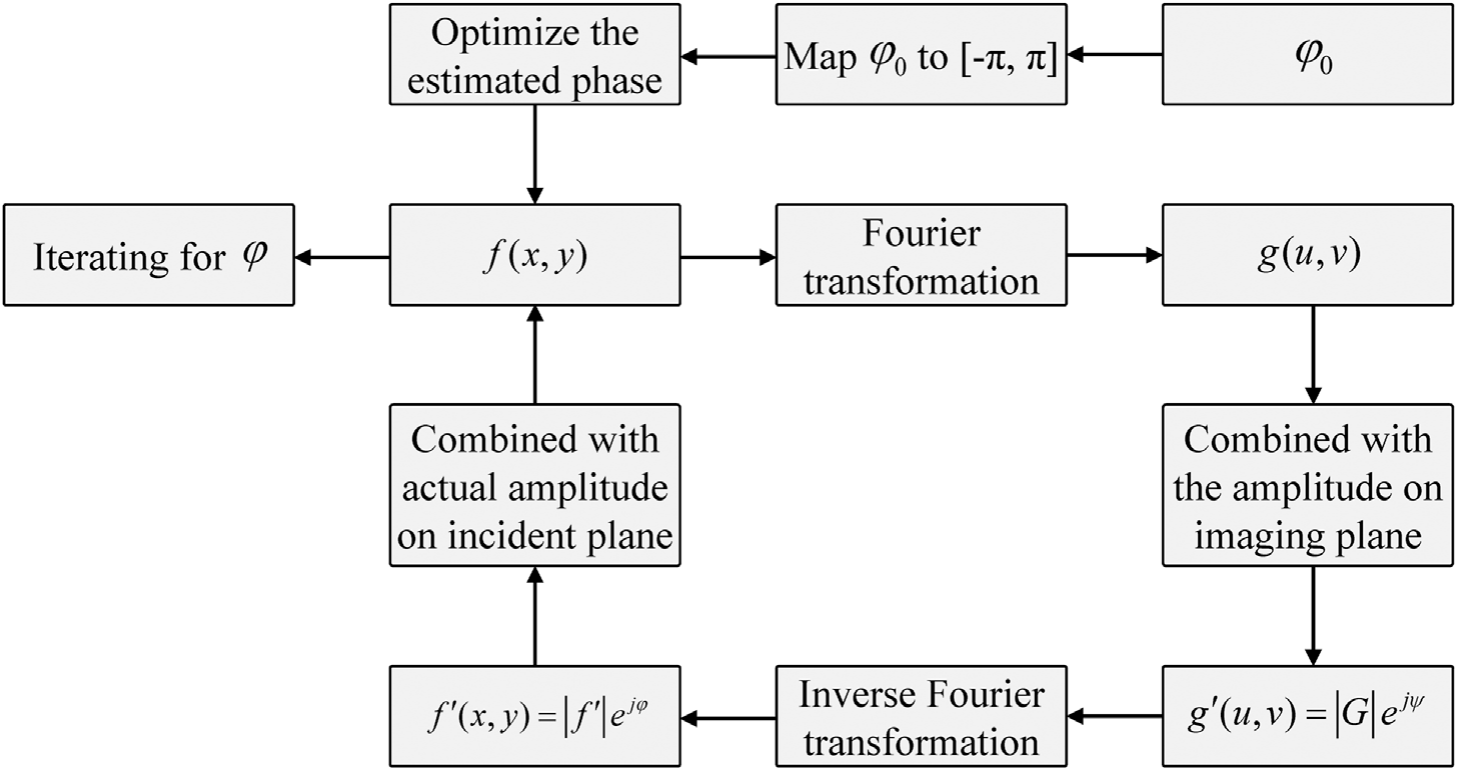
Schematic of the improved algorithm calculating steps.

The phase reconstruction algorithm is divided into two categories: The first interference phase detection category primarily includes the holographic technique and shear interference method, and the second non-interference detection category primarily includes the iterative method and transport-of-intensity equation method (Teague, 1983; Deutsch et al., 2008). The iterative method is based on the GS algorithm, reported by Gerchberg and Saxton (Gerchberg and Saxton, 1972), which is a typical algorithm to overcome the phase reconstruction problem. This algorithm adopts an iterative method for phase reconstruction by evaluating the relationship between the optical intensity distribution on the incident plane and that on the imaging plane. The GS method has a fast rate of convergence and high accuracy. In this section, we propose an improved GS algorithm, and Figure 2 demonstrates a schematic of holographic imaging with the improved GS algorithm. We normalize the phase and then set a threshold value of the processed phase to achieve a better reconstruction performance. Here, the optical field display on the incident plane using the wavefront coding technique is expressed as:

$$f\left(x,y\right)=\left|f\left(x,y\right)\right|e^{i\varphi \left(x,y\right)}$$ (4)

The optical field on the imaging plane using the wavefront coding technique is expressed as:

$$g\left(u,v\right)=\left|g\left(u,v\right)\right|e^{i\psi \left(u,v\right)}$$ (5)

where f(x,y) and g(u,v) demonstrate the amplitude of the optical field on the input plane and that on the imaging plane, respectively. φ(x,y) and ψ(u,v) demonstrate the phase on the input plane and imaging plane, respectively. The correlation between f(x,y) and g(u,v) can be expressed as follows:

f(x,y)=FFT{g(u,v)} (6)

A random generating phase of the optical field on the incident plane is input into the iterative operation as the initial phase, and the phase is continuously updated by the following iterations. Then, we normalize the phase and multiply the normalized phase by a factor value to optimize the initial phase. We combine g(u,v) with the amplitude on the imaging plane to obtain $g^{\prime }\left(u,v\right)$ . Inverse Fourier transformation is conducted on $g^{\prime }\left(u,v\right)$ to obtain $f^{\prime }\left(x,y\right)$ . The above steps are repeated to obtain the final phase.

### Comparative experimental models

In our study, an improved GS algorithm was adopted to reconstruct the wavefront phase map of urine sediment. Comparative experiments were conducted to validate the effectiveness of the proposed method. The performance of computer holography was analysed based on the phase and amplitude information of the cell reconstructed holographic image.

The urine sediment image was adopted as the experimental object. Computer holographic GS modelling is performed as follows: First, we perform preprocessing on an original RGB cell image, including downsampling the image with M × N pixels to an image with 256 × 256 pixels and conducting a greyscale operation. Second, the amplitude in each pixel was multiplied by a randomly generated phase value. Last, the iteration operation using the proposed method was conducted to obtain the reconstructed holographic cell image.

In this study, four models were built to investigate the effectiveness of the improved GS algorithm in cell holographic imaging. For Model 1, the original GS algorithm was adopted to obtain the amplitude information of the image, and the number of iterations was set to 20, 200, and 2,000. Then, we compared the amplitude of the holographic images with different iterations. For Model 2, the GS algorithm was adopted to obtain the phase information of the cell image. Neither Model 1 nor Model 2 was performed with phase mapping values. For Model 3, normalization processing and phase mapping were conducted on the amplitude and phase in Model 2, including transforming the image into greyscale from 0 to 1 and restricting the initial phase map value in the range of −π to π . This strategy could avoid the impact of phase deviation on the estimated accuracy. Then, we transformed the input image into an image with a complex format, and a random function was employed to generate a pair of images. The estimated phase was continuously calculated to approximate the actual phase in the iterative loop until the estimated phase was similar to the actual phase. The output estimated phase was mapped from [ −π , π ] to [0, 1]. The number of iterations was set to 20, 200 and 2,000 for comparison. Since the holographic algorithm takes the negative direction of the gradient of high-dimensional function as the maximum value, when the initial phase is not selected properly or the number of iterations is insufficient, the result will be very different from the optimal value. Thus for Model 4, β factor was introduced to restrict the initial value selection, and the phase matrix was normalized and then multiplied by the β factor value. The number of iterations was also set to 20, 200 and 2,000 for a horizontal comparison.

To quantitatively estimate the impact of the phase reconstruction, the root mean squared error (RMSE) is employed as an indicator to assess the results. The indicator of the RMSE is expressed as follows:

$$RMSE=\sqrt{\frac{1}{n}\overset{n}{\underset{i=1}{\sum }}{\left(y_{i}-y_{i}^{\prime }\right)}^{2}}$$ (7)

where n is the number of iterations, $y_{i}$ is the calculated phase of each iteration, and $y_{i}^{\prime }$ is the phase distribution of the last iteration.

## Results

**FIGURE 3:**
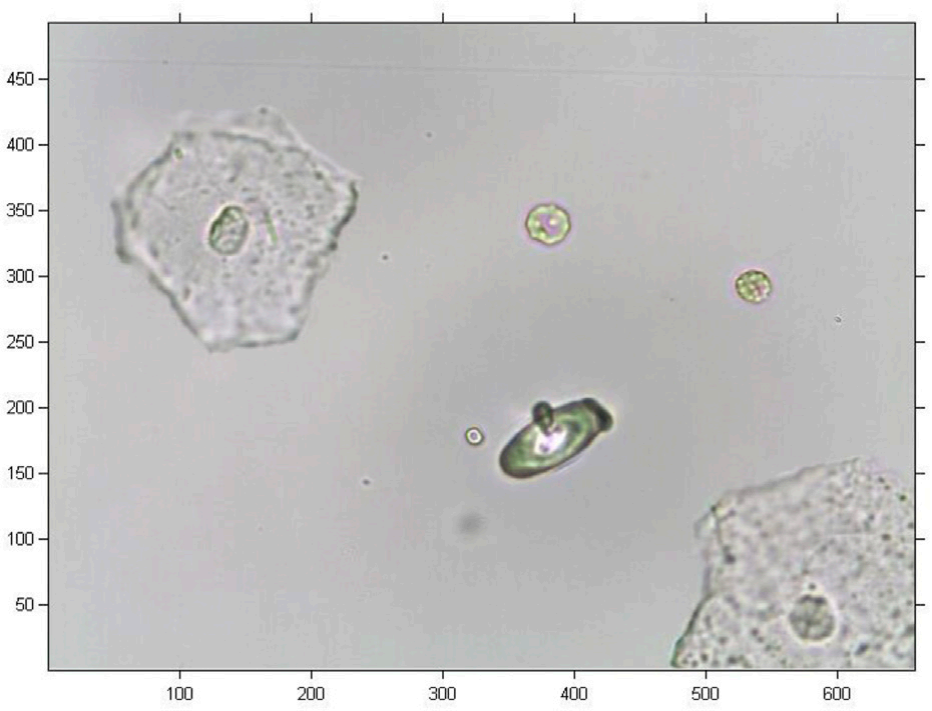
Original urine sediment image.

**FIGURE 4:**
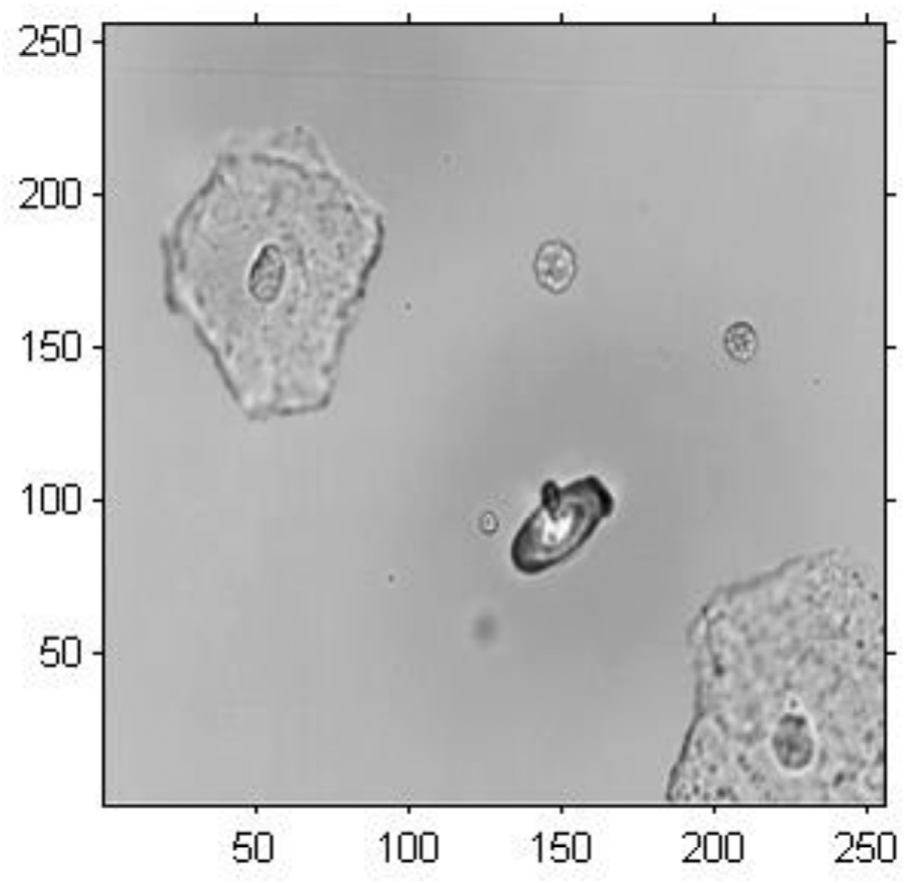
Pretreatment of image of urine sediment.

In this study, the original image was a coloured image of urine sediment with 493 × 659 pixels, as shown in Figure 3. Note that there are several epithelial cells and abnormal red blood cells in this image. There is a protruding cellular bleb on the membrane of the abnormal red blood cell. In order to extract the amplitude and phase information of the cell image, we conducted cropping and graying arithmetic on the original cell image. After the preprocessing the original image, a greyscale image with 256 × 256 pixels was obtained, as shown in Figure 4 .

**FIGURE 5:**
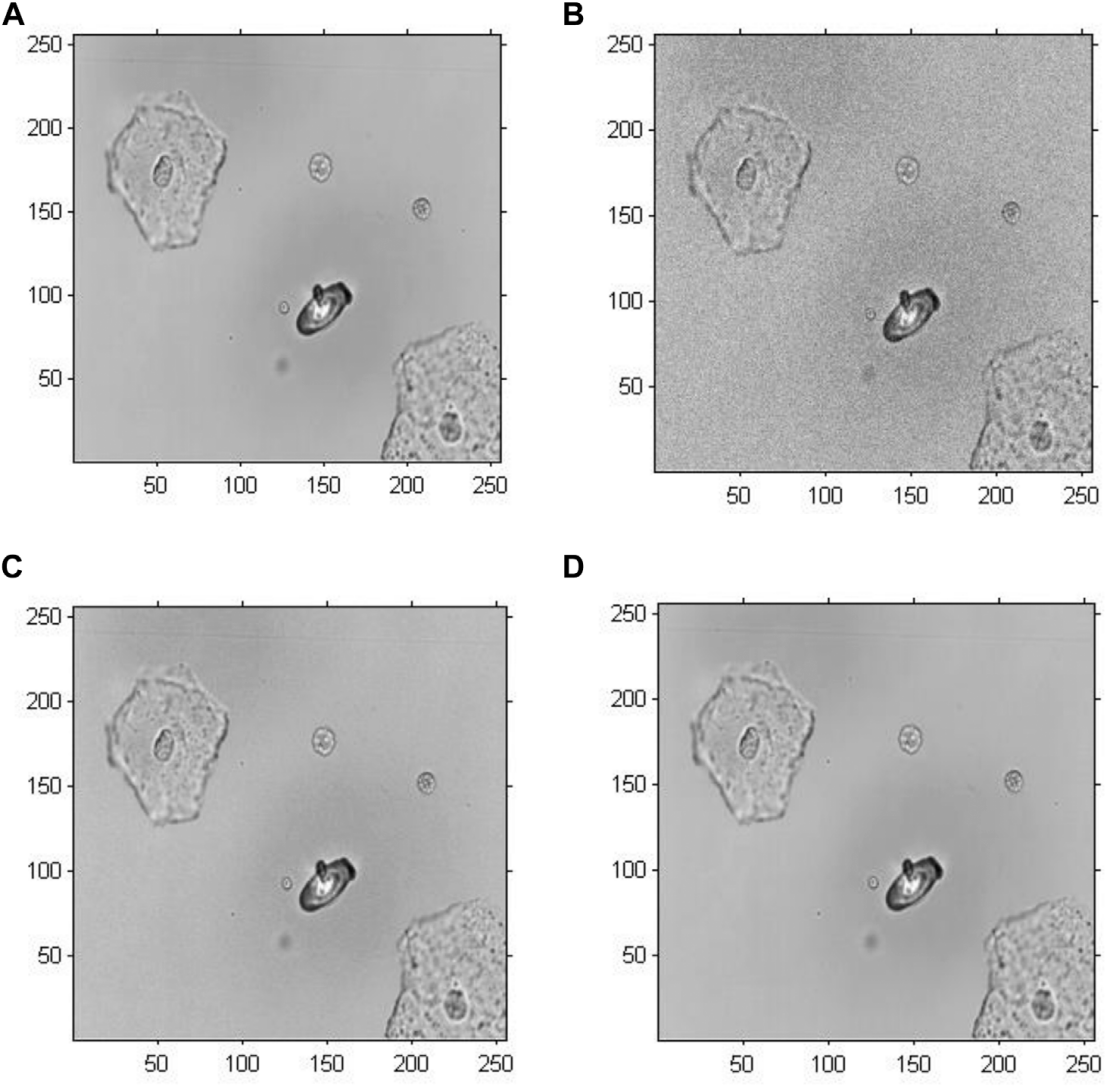
Amplitude reconstruction-based holographic image for Model 1. **(A)** Original greyscale image, **(B)** 20 iterative reconstruction images of amplitude information, **(C)** 200 iterative reconstruction images of amplitude information, and **(D)** 2000 iterative reconstruction images of amplitude information.

Figure 5 demonstrates the amplitude reconstruction-based holographic image for Model 1, which adopted the original GS algorithm to extract amplitude information with the number of iterations set to 20, 200 and 2,000. According to the visual comparison, there is a slight difference among the amplitude reconstruction-based holographic reconstructed images with different numbers of iterations. The reconstructed cell images only revealed a difference in brightness. Therefore, the amplitude information showed minimal impact on the cell holographic imaging.

**FIGURE 6:**
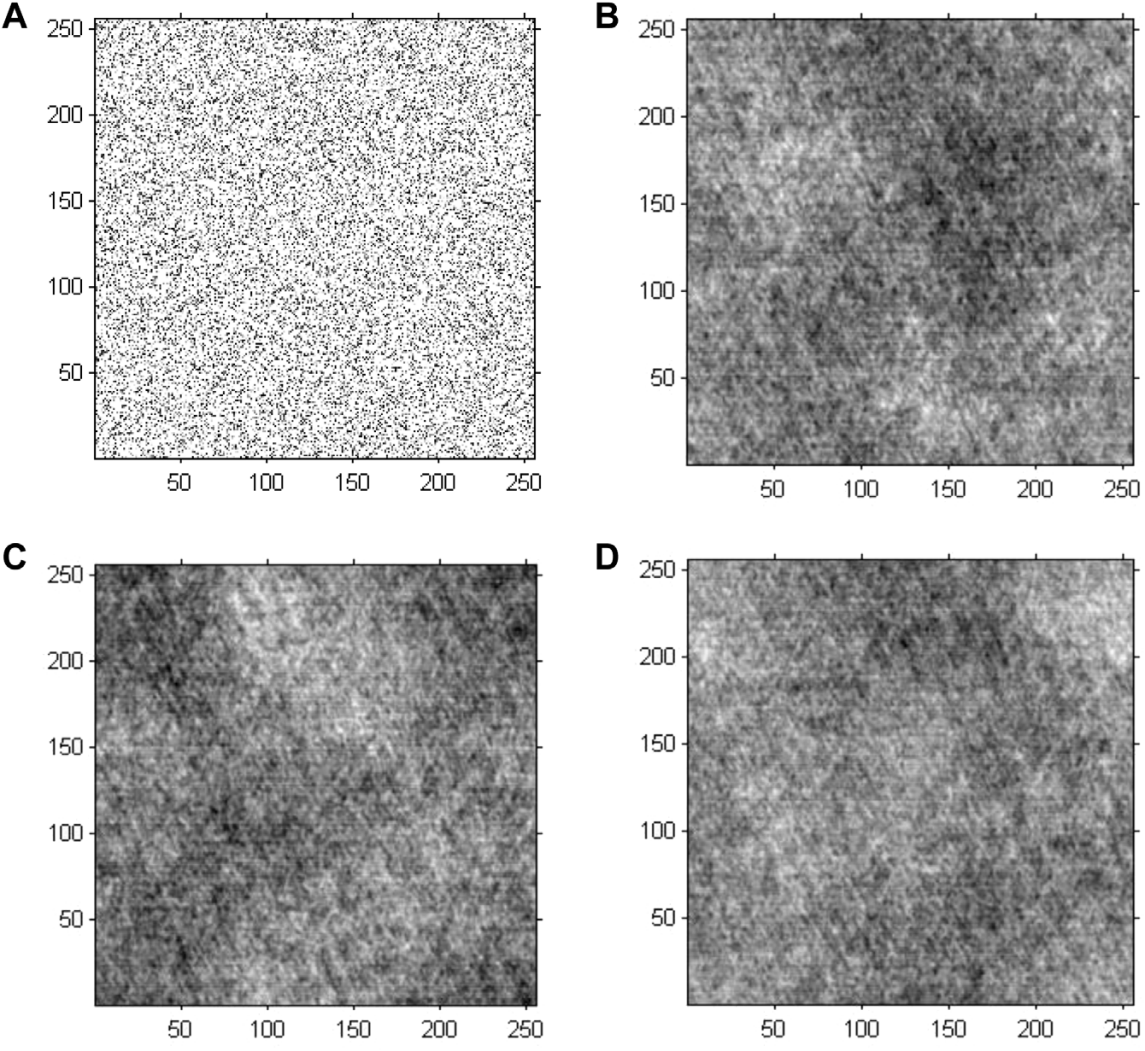
Phase reconstruction-based holographic image for Model 2 using the conventional GS algorithm. **(A)** Initial random phase, **(B)** 20 iterative reconstruction images of phase information, **(C)** 200 iterative reconstruction images of phase information, and **(D)** 2000 iterative reconstruction images of phase information.

The GS algorithm with a random initial phase was conducted on the preprocessed greyscale image to investigate the performance of cell holographic imaging. The phase reconstruction-based cell holographic images for Model 2 are shown in Figure 6. The comparative results demonstrated that the phase image would be fuzzy and chaotic without phase normalization, leading to different results for each calculation.

**FIGURE 7:**
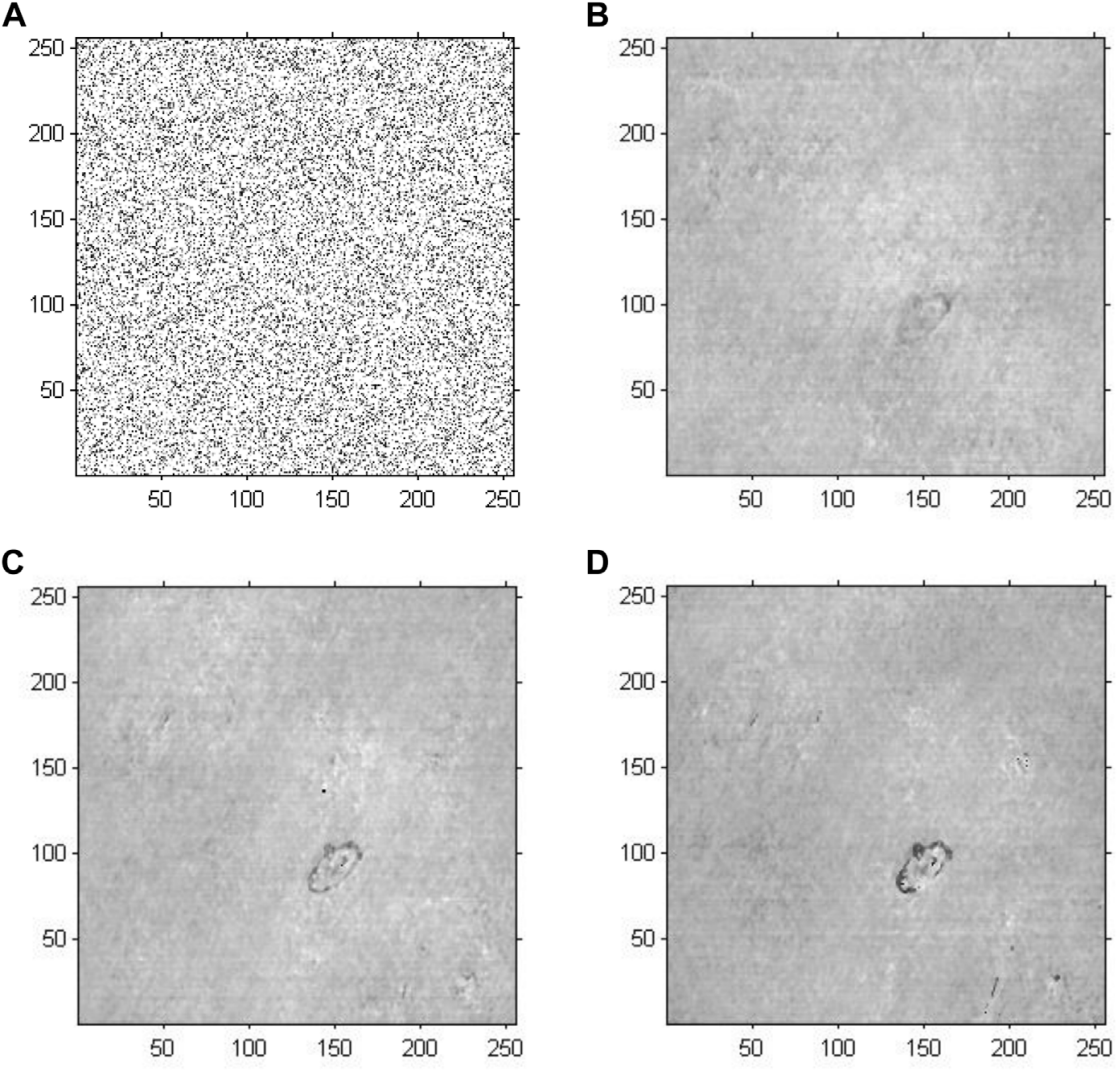
Phase reconstruction-based holographic image for Model 3 using the improved GS algorithm with the optimal initial phase. **(A)** Optimized initial phase. **(B)** Twenty iterative reconstruction images of phase information. **(C)** 200 iterative reconstruction images of phase information. **(D)** 2000 iterative reconstruction images of phase information.

Figure 7 demonstrates the phase reconstruction-based holographic image for Model 3, which adopted an improved GS algorithm with a random initial phase to extract phase information with the number of iterations set to 20, 200 and 2,000. According to the cell reconstructed images with different iterations, the comparative results suggested that as the number of iterations increased, the edges and the entire structure of abnormal red blood cells gradually became clearer, and the sharpness of the images increased. Compared with the results of Model 2, the abnormal blood red blood cells were successfully detected, and other impure cells were removed. Specifically, compared with the results of Model 1, the phase information had a more important role in cell holographic imaging than amplitude information, which only depicted the light and dark distribution.

**FIGURE 8:**
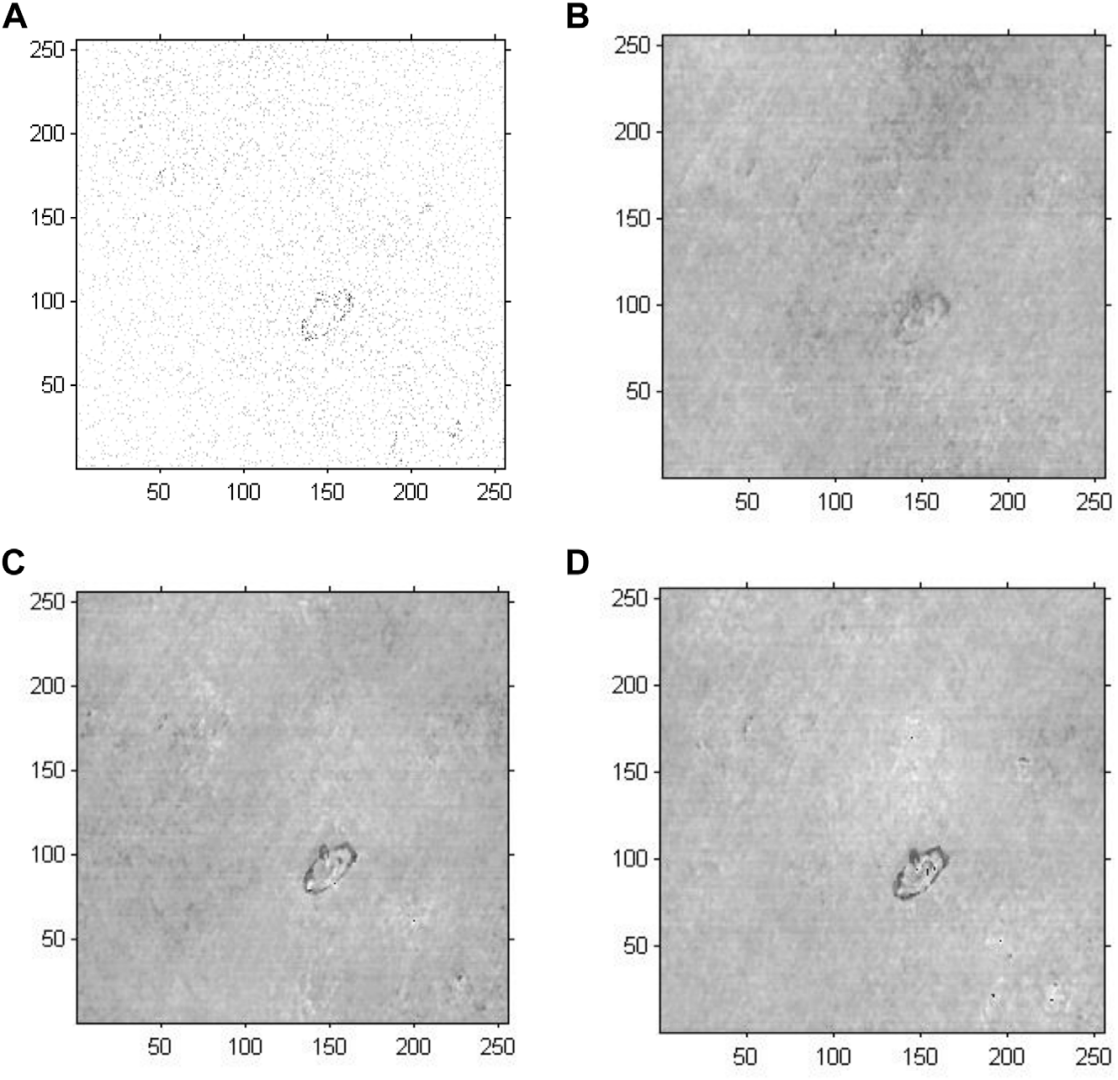
Phase reconstruction-based holographic image for Model 4 using the improved GS algorithm. **(A)** Optimized initial phase, **(B)** 20 iterative reconstruction images of phase information, **(C)** 200 iterative reconstruction images of phase information, and **(D)** 2000 iterative reconstruction images of phase information.

We conducted holographic imaging using an improved GS algorithm with an optimal initial phase to investigate the effectiveness of the proposed method for cell holographic imaging. Figure 8 shows the phase reconstruction-based cell holographic images for Model 4 with the initial phase limited by a certain threshold value and the number of iterations set to 20, 200 and 2,000. A comparison of the cell holographic image with different iterations reveals that the edges and the entire structure of abnormal blood red cells became clearer as the number of iterations increased. A comparison of the image results of Model 3 and Model 4 reveal that the abnormal blood red cell of Model 4 was clearer than the cell image of Model 3, which suggested that the cell holographic image using the proposed method showed effective performance in holographic reconstruction and cell detection.

The root mean squared error (RMSE) was calculated to quantitatively demonstrate the impact of phase reconstruction. We take the logarithm of the RMSE for easy comparison. The RMSE results support the above conclusion that the proposed method is suitable for cell holographic reconstruction.

Model 2 is the unmapped phase original GS algorithm; the phase peaks are disordered and small. Model 3 is the phase of the mapped original GS algorithm; the phase peak at the red blood cell in the target image is more obvious than that in Model 2. Model 4 is the phase of the mapped improved GS algorithm. Compared with Model 3, the phase peak at the position of the red blood cell is slightly higher, and the performance of the phase imaging image quality is slightly outstanding. Each model ultimately presents a phase energy diagram with 20, 200 and 2,000 iterations.

**FIGURE 9:**
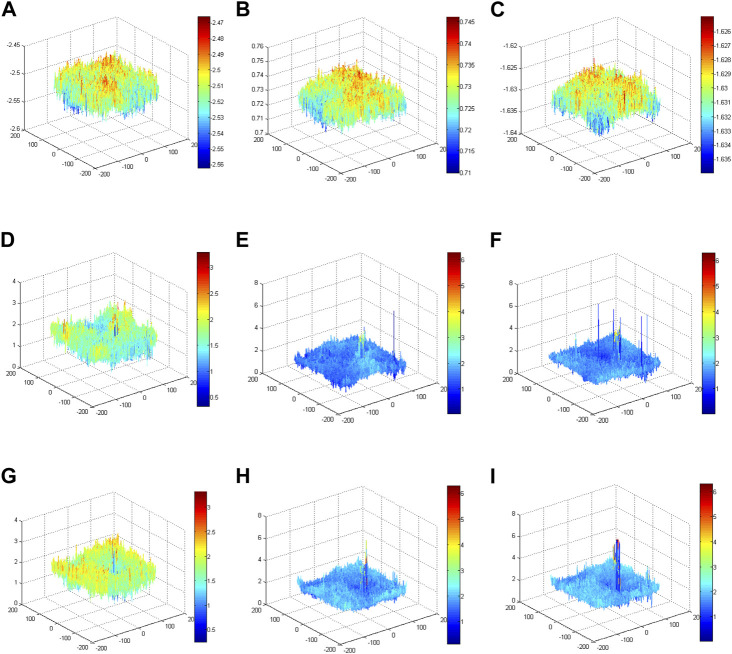
Phase energy map using the improved GS algorithm for different models. **(A)** Phase energy map with 20 iterations for Model 2. **(B)** Phase energy map with 200 iterations for Model 2. **(C)** Phase energy map with 2,000 iterations for Model 2. **(D)** Phase energy map with 20 iterations for Model 3. **(E)** Phase energy map with 200 iterations for Model 3. **(F)** Phase energy map with 2,000 iterations for Model 3. **(G)** Phase energy map with 20 iterations for Model 4. **(H)** Phase energy map with 200 iterations for Model 4. **(I)** Phase energy map with 2,000 iterations for Model 4.

To verify the enhancement effects of the improved GS algorithm, the phase distributions of different models were conducted using corresponding methods with different iterations, as shown in Figure 9. The phase results of Model 2 revealed that the phase distributions were scrambled and that the peak value was small. Notably, the phase peaks concentrated on the position of the blood red cell in Model 3 and Model 4. More specifically, the phase peaks of Model 4 at the position of the blood red cell were more concentrated and higher than the phase peaks in Model 3. These comparative results validated that the imaging quality using the proposed method was outstanding.

## Discussions

In this paper, we proposed an improved GS algorithm for cell reconstructed imaging, which demonstrated the capability of detecting normal red blood cells in urinary mussy circumstances. The proposed method adopted phase mapping and optimization to overcome the random and chaotic defects during conventional phase reconstruction processing.

The holographic image based on amplitude reconstruction results demonstrated that cell edges and detailed information is difficult to extract from the amplitude information. The comparative experimental results suggested that phase information was more important for cell holographic imaging than amplitude information. The amplitude information only demonstrates the light and shade distribution of the image, but does not associate with the content of cell characteristic properties, while the phase information is related to cell characteristic properties, such as edge and structure. According to the results of Model 3 and Model 4, when the phase was mapped in the range of −π to π , the impact of the phase deviation on the estimation accuracy could be avoided so that an accurate phase on the imaging plane was obtained. Phase normalization could render subsequent data processing more convenient and accelerate the convergence rate. As the greyscale value has a range, the real part value and imaginary part value of the Fourier transformation were unlimited. The mapping method was adopted to write the two matrices (amplitude and phase) into an image. The optimal initial phase could promote the accuracy of phase reconstruction with a small number of iterations. In addition, the correlation coefficient between the complex amplitude function and the initial input complex amplitude function after inverse transformation reduction was taken as a threshold. Setting constraints on iteration could decrease the iteration times of the GS algorithm and avoid the waste of computing resources.

Cells can be considered phase objects. As many cells have a low absorption or scattering rate of light, cell phase changes are closely associated with the structure and other morphological parameters. Therefore, digital holography has attracted more attention in the field of biological cell imaging as it is capable of quantitatively analysing biological cells and non-destructively and dynamically observing living cells. Specifically, Kemper and Von Bally (2008) applied digital holography technology in biomedical applications and carried out three-dimensional reconstruction of human liver tumour cells and red blood cells. Potcoava and Kim (2008) conducted research on the thickness of blood vessels and retina using digital holographic technology. Wang et al. (2016) promoted the resolution of digital holographic imaging by combining microspheres and image planes. Sandoz et al. (2019) adopted a digital holographic system to estimate the biochemical change in lipid droplets, which are responsible for lipid storage and metabolism. Research on variations in the physicochemical characteristics of the nucleolus in HeLa cells using a digital holographic system has been reported (Kim et al., 2019). Additionally, a report associated with the observation of living diatoms in seawater using a digital holographic system was presented (Umemura et al., 2020). In addition to quantitatively imaging single cells, many studies have referred to cell pathology monitoring using digital holographic techniques (Charrière et al., 2006; Sung et al., 2009; Hsu et al., 2012).

## Conclusion

In this paper, digital holographic technique could analyse cell physiological property without staining and dyeing, which is more suitable for the reconstruction of cell phase information, and makes up for the shortage that the traditional optical microscope can only provide amplitude images but is difficult to accurately distinguish the cell edges. An improved Gerchberg-Saxton algorithm was adopted to reconstruct the cell holographic image in light of the phase reconstruction information. Comparative experiments were carried out on four specific models with different phase constraint contidions, and the cell holographic image results clearly verified and revealed that this proposed improved GS method achieved better performance than the conventional GS method on the basis of phase reconstruction data. As the phase image reveals not only the spatial information but also the temporal statistics during the transmission of various frequency waves, the accumulation of phase information is able to depict the accurate shape parameters of the recorded object. Then, validation experiments were conducted on urine sediment images, and the reconstructed holographic image results demonstrated that this improved GS method was able to extract abnormal red blood cells with sharp edges and filter out other impure objects. In conclusion, the reconstructed holographic phase image achieved by the proposed method had a high signal-to-noise ratio, and the method was specific to discriminate the morphological parameters. Thus, these results demonstrated the possibility of this proposed holographic method in practical biomedical applications in cell activities monitoring.

## Data Availability

The original contributions presented in the study are included in the article/supplementary material, further inquiries can be directed to the corresponding authors.
